# Repetitive Low-Level Blast Exposure Alters Circulating Myeloperoxidase, Matrix Metalloproteinases, and Neurovascular Endothelial Molecules in Experienced Military Breachers

**DOI:** 10.3390/ijms26051808

**Published:** 2025-02-20

**Authors:** Shawn G. Rhind, Maria Y. Shiu, Catherine Tenn, Ann Nakashima, Rakesh Jetly, Venkata Siva Sai Sujith Sajja, Joseph B. Long, Oshin Vartanian

**Affiliations:** 1 Defence Research and Development Canada, Toronto Research Centre, Toronto, ON M3K 2C9, USA; maria.shiu@ecn.forces.gc.ca (M.Y.S.); oshin.vartanian@ecn.force.gc.ca (O.V.); 2 Faculty of Kinesiology and Physical Education, University of Toronto, Toronto, ON M5S 2W6, Canada; 3 Defence Research and Development Canada, Suffield Research Centre, Medicine Hat, AB T1A 8K6, Canada; catherine.tenn@drdc-rddc.gc.ca; 4 The Institute of Mental Health Research, University of Ottawa, Royal Ottawa Hospital, Ottawa, ON K1Z 7K4, Canada; rakesh@drjetly.com; 5 Blast-Induced NeuroTrauma Branch, Walter Reed Army Institute of Research, Silver Spring, MD 20910, USAjoseph.b.long.civ@mail.mil (J.B.L.); 6 Department of Psychology, University of Toronto, Toronto, ON M5S 1A1, Canada

**Keywords:** blast-induced neurotrauma, blood–brain barrier, myeloperoxidase, matrix metalloproteinases, neurovascular unit, endothelium, tight junction proteins, aquaporin, occludin, syndecan

## Abstract

Repeated exposure to low-level blast overpressure, frequently experienced during explosive breaching and heavy weapons use in training and operations, is increasingly recognised as a serious risk to the neurological health of military personnel. Although research on the underlying pathobiological mechanisms in humans remains limited, this study investigated the effects of such exposure on circulating molecular biomarkers associated with inflammation, neurovascular damage, and endothelial injury. Blood samples from military breachers were analysed for myeloperoxidase (MPO), matrix metalloproteinases (MMPs), and junctional proteins indicative of blood–brain barrier (BBB) disruption and endothelial damage, including occludin (OCLN), zonula occludens-1 (ZO-1), aquaporin-4 (AQP4), and syndecan-1 (SD-1). The results revealed significantly elevated levels of MPO, MMP-3, MMP-9, and MMP-10 in breachers compared to unexposed controls, suggesting heightened inflammation, oxidative stress, and vascular injury. Increased levels of OCLN and SD-1 further indicated BBB disruption and endothelial glycocalyx degradation in breachers. These findings highlight the potential for chronic neurovascular unit damage/dysfunction from repeated blast exposure and underscore the importance of early targeted interventions—such as reducing oxidative stress, reinforcing BBB integrity, and managing inflammation—that could be essential in mitigating the risk of long-term neurological impairment associated with blast exposure.

## 1. Introduction

Repeated occupational exposure to low-level blast overpressure, as encountered during explosive breaching, heavy weapons use, and other military operations, poses serious risks to service members’ brain health and performance [1,2,3,4]. Although high-intensity blasts often produce overt hemorrhage and gross tissue disruption, growing evidence suggests that milder or subclinical exposures can also initiate and progressively worsen cerebrovascular dysfunction, ultimately compromising the integrity of the central nervous system (CNS) [5,6,7,8,9]. Studies involving military breachers have consistently identified detrimental outcomes linked to repetitive blasts, including self-reported symptoms, neurocognitive deficits, neuroimaging abnormalities, and altered molecular biomarker profiles [10,11,12,13,14,15,16,17,18,19,20,21]. These injuries involve mechanical deformation of blood vessels, transient or persistent blood–brain barrier (BBB) disruption, vasospasm, and microhemorrhages, all of which may remain subclinical but accumulate over time [9,22,23,24,25,26,27,28]. Findings from both clinical and experimental work indicate a continuum of blast-related pathophysiology, wherein repeated low-intensity insults can induce immediate subclinical disturbances that progress to overt and persistent neurological damage [29,30,31,32,33,34,35]. Each additional exposure appears to heighten the brain’s vulnerability to further injury and diminish its recovery capacity [11,17,36,37], placing those who experience frequent blasts at a substantially increased risk of chronic functional deficits and eventual progression to neurodegenerative conditions [38,39].

The mechanisms underlying the pathological effects of low-level primary blast shockwaves on the nervous system remain incompletely understood [40,41,42,43]. Nonetheless, accumulating evidence indicates that the brain’s vasculature is particularly vulnerable to repeated blast exposure and may play a central role in blast-induced neuropathology [23,44,45,46,47,48]. Vascular insults can arise through direct transcranial transmission of shockwaves—where rapid acceleration and deformation compromise the cerebral microvasculature, BBB, and neurovascular unit (NVU)—as well as through indirect thoracic mechanisms, wherein blast-induced intrathoracic pressure surges propagate hydrodynamic forces to the brain [40,49,50,51,52,53,54,55]. The NVU, comprising vascular cells (endothelial cells, pericytes, glycocalyx, tight junctions), glial cells (astrocytes, microglia, oligodendrocytes), neurons, and the extracellular matrix (ECM), is essential for regulating cerebral blood flow, maintaining BBB function, and preserving neuronal homeostasis [27,56,57,58]. The mechanical disruption of these components triggers a cascade of molecular and cellular responses, including oxidative stress and neuroinflammation, which can amplify BBB breakdown and accelerate neurodegenerative processes [8,25,59,60].

Experimental rodent models of repeated low-level primary blast exposure demonstrate extensive microvascular damage, compromised BBB integrity, and increased vascular permeability [23,50,54,61,62,63,64,65]. Under normal conditions, the BBB selectively restricts harmful substances from entering the brain; however, repetitive blast-induced disruptions allow neurotoxic molecules and immune cells to cross into the parenchyma, precipitating vasogenic edema, ischemia, and heightened neuroinflammation [66,67,68]. Among the principal molecular contributors to these vascular and inflammatory effects are reactive oxygen species (ROS), myeloperoxidase (MPO), and matrix metalloproteinases (MMPs) [8,9,69,70,71,72,73]. MPO, released by activated neutrophils and other immune cells, intensifies oxidative stress and proinflammatory signaling, thereby exacerbating neurovascular damage [74,75]. Meanwhile, MMPs degrade ECM and tight junction proteins, including occludin (OCLN), claudin-5 (CLDN5), and zonula occludens-1 (ZO-1), further destabilising the BBB and increasing endothelial permeability [24,76,77,78,79,80,81,82,83]. By facilitating the breakdown of critical structural barriers, these enzyme-driven processes not only magnify acute injuries but also establish a pathway toward chronic neurodegenerative changes.

Aquaporin-4 (AQP4), an astrocytic water channel that regulates fluid homeostasis and underpins the glymphatic system, is among the key neurovascular molecules implicated in repeated blast exposure [84,85]. Disruptions in AQP4 expression and localisation, along with enlargement of perivascular spaces and impaired glymphatic clearance, can lead to cerebral edema, elevated intracranial pressure, and heightened neuroinflammation, thereby exacerbating brain tissue damage and accelerating the risk of chronic neurodegeneration [63,67,86,87,88,89]. Similarly, syndecan-1 (SD-1)—a heparan sulfate proteoglycan essential for endothelial glycocalyx integrity, vascular tone regulation, and solute exchange—plays a critical role in preserving barrier function [90,91,92]. Following repetitive head trauma, SD-1 can be cleaved by MMPs, eroding the protective luminal surface of blood vessels and contributing to endothelial dysfunction, vascular inflammation, and compromised BBB permeability [26,93,94,95,96]. Chronic alterations in AQP4 and SD-1 thereby form key molecular links between acute neurovascular injuries and the eventual onset of neurodegenerative pathology.

Over time, repeated blast exposure can induce cumulative molecular and cellular perturbations that drive chronic vascular pathology, intensify inflammation, and weaken the BBB, ultimately setting the stage for progressive neurodegenerative processes and cognitive decline [8,35,58]. As a result, military personnel who encounter frequent low-level blasts face an elevated risk of enduring neurological disorders, including chronic traumatic encephalopathy (CTE) and other proteinopathies [97,98,99,100]. Elucidating the biomolecular pathways that underlie these injuries and identifying reliable, translatable biomarkers of early neurovascular damage remain critical for enabling timely diagnosis and developing targeted interventions that protect against the latent effects of repeated blast exposure [21,101,102].

In our prior investigations of military breachers routinely subjected to low-level blasts, we documented persistent post-concussive symptoms, impaired cognitive-motor integration, and diminished self-reported physical and mental health when compared with unexposed individuals [17,103]. Building on these clinical and functional findings, this present study examines blood samples from the same cohort to assess molecular mediators associated with blast-induced neurovascular insults and BBB disruption. We hypothesised that individuals with higher cumulative blast exposure would show evidence of ultrastructural damage to the cerebral vasculature and NVU, reflected by elevated levels of inflammatory enzymes (MPO, MMPs), tight junction proteins (OCLN, ZO-1), the astrocytic water-channel protein AQP4, and glycocalyx-derived endothelial injury marker SD-1, all of which would indicate an increased risk for progressive neurodegeneration.

## 2. Results

### 2.1. Participant Demographics and Military Characteristics

The demographic, service history, and military occupational exposure characteristics for the blast-exposed Canadian Forces School of Military Engineering (CFSME) breacher instructors and range staff (n = 18; referred to as “Breachers”) and the unexposed Canadian Armed Forces (CAF) “Controls” (n = 19) are summarised in Table 1. Overall, the two groups were well matched in terms of age (median: 32 years, range: 22–52), sex (89.5% male), education, lifestyle, and injury history.

Breachers reported significantly more years of military service (Cohen’s D = 3.5, *p* < 0.001), years of breaching experience (Cohen’s D = 3.5, *p* < 0.001), and higher rates of combat deployment (Cohen’s D = 2.9, *p* < 0.001) compared to CAF controls. Senior NCMs comprised a greater proportion of breachers/range staff (Cohen’s D = 2.6, *p* < 0.001), while CAF controls included more Junior NCMs (Cohen’s D = –1.3, *p* = 0.004). Breachers had substantially higher career exposure to explosive blasts compared to the controls.

### 2.2. History of Head Trauma

Table 2 indicates no significant differences in head trauma history between groups, except for blast exposure, which was universal among breachers (100%) but occurred in only 10.2% of the controls (Cohen’s D = 6.3, *p* < 0.001). No differences in self-reported concussion history were observed between groups.

### 2.3. Neuropsychological and Neurocognitive Measures

Table 3 highlights key differences in neuropsychological and neurocognitive measures comparing breachers to the controls. Breachers scored significantly lower on the SF-36 Energy subscale (Cohen’s D = –0.99, *p* = 0.022). Rivermead Post-Concussion Questionnaire (RPQ) results showed higher scores in breachers for early post-concussive symptoms (RPQ-3; Cohen’s D = 1.53, *p* < 0.001) and late post-concussive symptoms (RPQ-13; Cohen’s D = 1.74, *p* < 0.001). Breachers also had elevated scores in somatic (Cohen’s D = 1.48, *p* = 0.004), cognitive (Cohen’s D = 1.46, *p* = 0.004), and emotional symptoms (Cohen’s D = 1.52, *p* < 0.001) compared to the controls. These findings highlight significant neuropsychological and service-related differences between the breachers and controls, with the breachers demonstrating greater post-concussive symptoms and energy deficits, likely reflecting the cumulative impact of blast exposure.

The neurocognitive battery assessed performance across four tasks, each targeting distinct cognitive domains. The delayed matching-to-sample (dMTS) test evaluated short-term visual memory and pattern recognition [104], with accuracy measured as the percentage of correct responses (out of 25 trials). The four-choice reaction time task (four-choice RT task) assessed rapid and accurate responses to simple visual stimuli presented on a screen [105], using reaction times for correct responses as the dependent variable. The Stroop task measured executive function, specifically inhibition [106], by comparing reaction times for identifying the colour of incongruent versus congruent word trials. The n-back task, a test of working memory requiring the maintenance and updating of dynamic rehearsal sets [107], used sensitivity (d′) as the dependent variable, with higher positive values indicating better sensitivity and negative values reflecting judgement errors. None of these metrics showed sensitivity to blast effects. However, the controls exhibited significantly higher failure rates in memory and comprehension tests compared to breachers (Cohen’s D = 0.91, *p* < 0.001).

### 2.4. Circulating Myeloperoxidase and Matrix Metalloproteinases in Breachers and Controls

**Figure 1:**
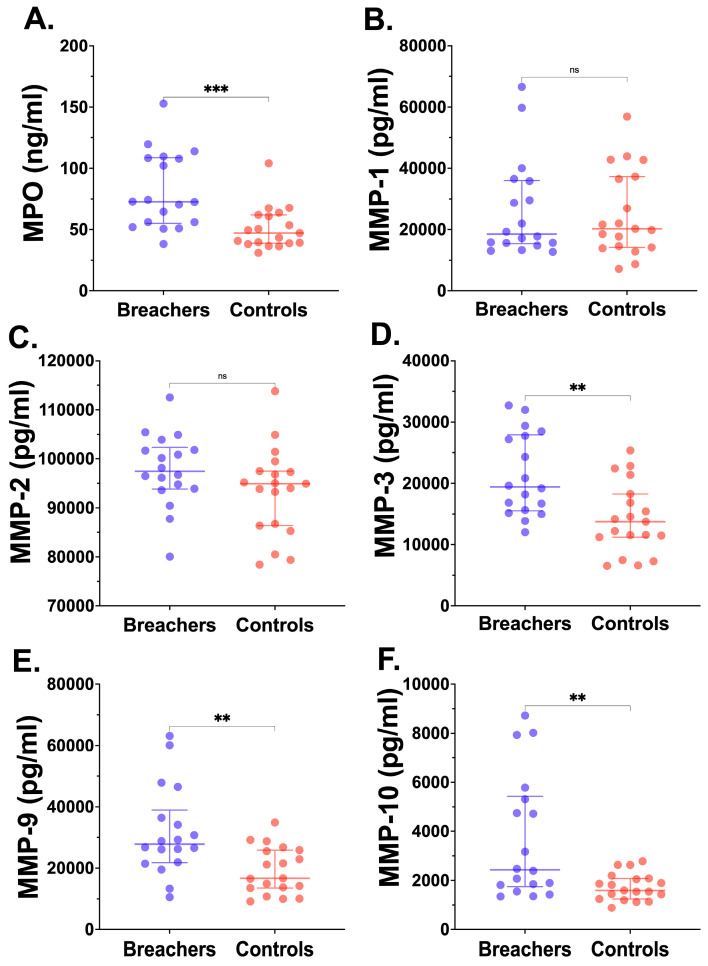
Plasma myeloperoxidase (MPO) and matrix metalloproteinases (MMPs) in breachers vs. controls. Data are displayed as individual scatter plots showing the median and interquartile range (IQR; bars) for each biomarker among blast-exposed breachers (closed blue circles) and unexposed controls (closed red boxes), as indicated; MPO (**A**), MMP-1 (**B**), MMP-2 (**C**), MMP-3 (**D**), MMP-9 (**E**), and MMP-10 (**F**). Mann–Whitney *U*-test significance breachers vs. controls: ns (not significant), ** *p* < 0.01, *** *p* < 0.001.

Proinflammatory MPO and five MMPs, which degrade and remodel proteins like elastin and collagen, were measured in plasma samples from all participants. As illustrated in Figure 1A–F, MPO and MMP-3, MMP-9, and MMP-10 concentrations were significantly elevated in breachers compared to the controls. Specifically, MPO levels in Breachers had a median of 72.5 compared to 47.2 in the controls (IQR: 152.8–55.0 vs. 38.9–61.9, respectively). MMP-3 (median: 19,402 vs. 13,718; IQR: 15,531–32,710 vs. 11,209–25,339), MMP-9 (median: 27,781 vs. 16,694; IQR: 21,782–38,961 vs. 13,517–25,863), and MMP-10 (median: 2431 vs. 1593; IQR: 1750–5429 vs. 1246–2080) were all notably higher in breachers than the controls. No significant differences were found for MMP-1 and MMP-2 between the two groups.

### 2.5. Circulating Neurovascular Injury Molecules in Breachers and Controls

**Figure 2:**
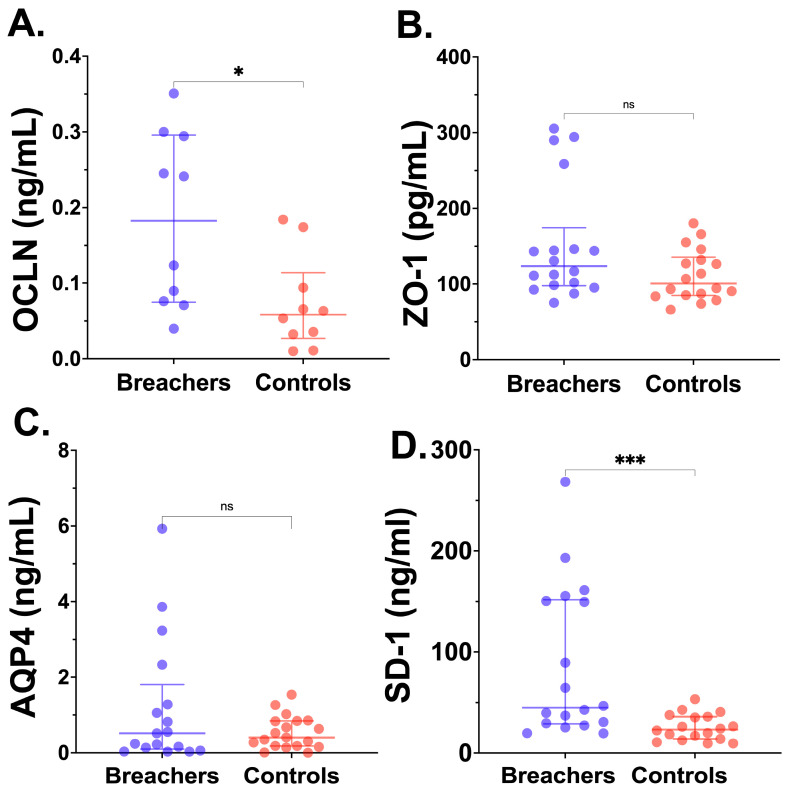
Plasma neurovascular molecules in breachers vs. controls. Data are displayed as scatter plots, showing the median and interquartile range (IQR; bars) for each biomarker in blast-exposed breachers (closed blue circles) and unexposed controls (closed red circles); occludin (OCLN) (**A**), zonula occludens-1 (ZO-1) (**B**), aquaporin-4 (AQP4) (**C**), syndecan-1 (SD-1) (**D**). Mann–Whitney *U*-test significance breachers vs. controls: ns (not significant), * *p* < 0.05, *** *p* < 0.001.

Key NVU molecular constituents that underly BBB integrity and vascular endothelial damage were detectable in the majority of participants. As displayed in Figure 2A–D, breachers had significantly elevated plasma levels of the TJ protein OCLD and SD-1, a marker of endothelial glycocalyx degradation, compared to the controls. However, while breachers had higher levels of ZO-1 and aquaporin-4 (AQP4) overall, these did not differ significantly between groups.

## 3. Discussion

Exposure to explosive blast overpressure waves, a frequent occurrence in military settings, has been increasingly associated with significant effects on the brain and CNS [3]. The neurovascular unit plays a pivotal role in preserving the integrity of the BBB, which shields the brain from harmful substances. Following blast exposure, however, the BBB is often compromised, leading to various neuropathological sequelae. Research indicates that blast waves exert both mechanical stress and biochemical changes that specifically disrupt tight junction proteins, thereby weakening the BBB’s selective barrier [83]. This injury response is largely driven by elevated inflammatory and degradative enzymes, most notably MPO and MMPs [8,9].

In this cross-sectional cohort study, military breachers with extensive low-level blast exposure displayed neurovascular and BBB disruption, evidenced by higher plasma levels of MPO, MMP-3, MMP-9, MMP-10, OCLN, and SD-1 compared with unexposed controls. These biomarker elevations reflect pathways of inflammation, oxidative stress, and endothelial injury and underscore the cumulative impact of occupational blast exposure on brain health. The findings point to a heightened risk of long-term neurodegenerative and cognitive sequelae in chronically exposed individuals, emphasising the importance of monitoring these biomarkers to assess neurovascular damage and guide protective strategies in blast-exposed populations.

### 3.1. Blast-Induced Myeloperoxidase Expression, Oxidative Stress, and Neuroinflammation

Blast-induced trauma activates MPO, which generates ROS and amplifies oxidative stress within the brain. Released by activated neutrophils and microglia, MPO catalyses the production of ROS, including hydrogen peroxide and superoxide anion, initiating a potent oxidative burst that disrupts BBB structure and increases its permeability [74,79,108]. This oxidative stress damages cellular components such as lipids, proteins, and DNA, exacerbating neuronal injury and impairing NVU and BBB integrity [75,109,110]. Although MPO activation may initially serve as a protective response, its repeated activation post-injury creates a self-sustaining cycle of oxidative stress and inflammation, leading to significant neurotoxic effects on BBB endothelial cells that progressively undermine vascular stability and contribute to long-term neurovascular deterioration [81,109,111].

The recent literature provides a framework for understanding how repeated blast exposure leads to progressive neurovascular injury. Animal studies identify MPO as a marker of oxidative stress and inflammation following primary blast wave exposure [48,51,112], supporting our findings of elevated MPO levels in breachers subjected to repeated low-level blasts. In rodent models, repeated blasts significantly upregulate MPO in the brain and other organs, which correlates with increased ROS production, BBB disruption, and neuroinflammatory responses [23,54,71,72,113,114,115,116]. Cernak et al., for example, observed a pronounced increase in systemic MPO activity through in vivo bioluminescence imaging of activated neutrophils in the brain, lungs, and gastrointestinal tract of mice following low-intensity blast exposure [70]. Notably, brain MPO levels peaked at 30 days post-injury, even when head protection was provided, suggesting that systemic inflammatory cells infiltrate the CNS. This sustained MPO elevation likely disrupts the BBB, enabling peripheral immune cells and neurotoxic substances to penetrate the brain parenchyma, thereby intensifying neuroinflammation and oxidative damage [109,117]. Valiyaveettil et al. also found that repeated blast exposure induces systemic inflammation, resulting in elevated plasma MPO activity and cerebral vasoconstriction, highlighting the role of systemic factors in exacerbating brain injury [118,119]. Similarly, Rodriguez et al. demonstrated that repeated low-level blast exposure elevates NADPH oxidase and MPO, contributing to cumulative NVU damage and associated neurobehavioural deficits [120].

Moreover, these findings are consistent with studies by Chavko et al., who found that moderate blast overpressure in rats leads to lung and endothelial injury characterised by elevated MPO and oxidative damage, with recovery aided by antioxidant enzymes like heme oxygenase-1 and superoxide dismutase [121]. Further research determined that N-acetylcysteine amide effectively reduces MPO-driven inflammation and oxidative stress, suggesting its potential to mitigate both pulmonary and neurological injuries following the blast and supporting antioxidant therapies that target MPO [122,123,124]. Although direct measurements of MPO in blast-exposed human populations are limited, the patterns observed in animal models and other forms of brain injury offer valuable insights [125,126,127]. The elevated plasma MPO levels observed in breachers align with these findings, suggesting that similar oxidative and inflammatory mechanisms may be active in blast neurotrauma and underscoring that cumulative blast exposure likely amplifies neurovascular damage and progressively compromises the BBB through sustained oxidative stress [3,8,9,24,50].

### 3.2. Blast-Induced Upregulation of Matrix Metalloproteinases and ECM Degradation

Blast exposure markedly upregulates MMPs, a versatile family of zinc-dependent endopeptidases crucial for ECM remodelling and BBB integrity maintenance [56,78]. While MMPs support synaptic plasticity, tissue repair, and neurogenesis under normal conditions [128,129,130], neurotrauma activates specific MMPs through MPO-generated ROS and inflammatory signals [81,131,132]. This activation converts MMP-2 (Gelatinase A), MMP-3 (Stromelysin-1), MMP-9 (Gelatinase B), and MMP-10 (Stromelysin-2) from inactive zymogens into active proteases that selectively degrade essential ECM structural proteins and BBB tight junction components [79,133]. Specifically, MMP-2 primarily cleaves type IV collagen in basement membranes, contributing to BBB weakening [77]. MMP-3 degrades a broad spectrum of ECM components, including fibronectin and laminin, while also activating other MMPs, thus amplifying proteolytic activity. MMP-9 targets type IV collagen and additional BBB-crucial structural proteins, further increasing vascular permeability and neuroinflammation [128,134]. MMP-10 exhibits functional overlap with MMP-3 but is particularly involved in modulating inflammatory responses and enhancing leukocyte infiltration, thereby exacerbating BBB disruption and neurovascular injury [66,135,136]. This coordinated enzymatic activity destabilises the NVU by degrading ECM support structures and weakening endothelial cell tight junctions [50,54,67,68]. The resulting self-sustaining cycle of tissue damage progressively compromises the NVU with successive blasts, potentially leading to long-term neurological consequences [8,137].

Our study revealed significant elevations in circulating levels of MMP-3, MMP-9, and MMP-10 among blast-exposed breachers compared to the controls, underscoring the central role of MMP activation in response to blast exposure [8,50,138]. These elevated MMP levels align with their established functions in BBB disruption, neuroinflammation, and ECM degradation, highlighting the cumulative neurovascular damage resulting from repeated blast exposure and its potential contribution to long-term brain health risks. MMP-9, a key mediator of acute BBB permeability, has been extensively documented in animal models of blast-induced neurotrauma. Its upregulation is associated with BBB breakdown, increased permeability to peripheral immune cells, edema, neuronal injury, and potential neurodegenerative changes [113,139,140,141]. Kuriakose et al. demonstrated that blast exposure significantly elevated superoxide production and activated MMP-3 and MMP-9 in rat brain cell lysates, resulting in BBB disruption within hours post-blast. Notably, pre-treatment with the NOX inhibitor apocynin effectively blocked MMP upregulation, preserved tight junction proteins, and reduced BBB permeability [71]. Kawoos et al. reported rapid MMP-9 upregulation in brain tissue and plasma following moderate blast exposure in rats, correlating with early BBB permeability changes and highlighting MMP-9’s pivotal role in mediating neurovascular injury after blast trauma [54]. In a thoracic blast injury mouse model, Cong et al. found that elevated MMP-9 exacerbated BBB disruption and oxidative stress, with particularly severe effects in DDAH1-deficient mice [142]. Furthermore, Gama Sosa et al. identified the chronic upregulation of MMP-9 and MMP-2 in a rat blast model, resulting in progressive vascular remodelling and sustained neuroinflammation [72]. The roles of MMP-3 and MMP-10 in amplifying ECM breakdown and inflammatory cascades further emphasise the multifaceted nature of blast-induced neurovascular damage.

Human studies further support these findings, linking elevated MMP-9 levels to BBB disruption and neuroinflammation in blast-exposed military personnel [11]. Notably, Agoston et al. observed that repeated subconcussive blast exposure in military trainees led to elevated circulating MMP-9 and other biomarkers of neurovascular injury, inflammation, and neuronal damage, with MMP-9 remaining high for up to three months post-exposure [20]. This prolonged elevation reinforces MMP-9’s role in ongoing neurovascular damage and inflammation due to low-level blast exposure. Ravin et al. also found that simulated blast exposure in cultured human CNS cells increased MMP-9 expression, linking it to astrocyte-mediated neuroinflammatory signaling [143]. Our findings of sustained MMP-3, MMP-9, and MMP-10 upregulation in breachers suggest that repeated low-level blasts activate a broader spectrum of MMPs, compounding NVU damage and elevating the risk of neurodegeneration [144,145]. While MMP-9 is a key mediator of acute BBB permeability, MMP-3 and MMP-10 contribute to ECM degradation and amplify inflammatory cascades, respectively [78,133]. The absence of significant changes in MMP-1 and MMP-2 may reflect their primary roles in baseline ECM maintenance, processes less affected compared to the acute inflammatory and BBB-disruptive activities driven by MMP-3, MMP-9, and MMP-10 [132,140].

These results highlight MMP-3, MMP-9, and MMP-10 as key biomarkers of blast-related neurovascular injury and potential therapeutic targets for preserving BBB integrity [129]. The complex interplay between these MMPs, oxidative stress, and BBB integrity reveals the multifaceted nature of blast-induced neurotrauma, elucidating mechanisms underlying both acute and chronic neurological damage. Preclinical studies suggest that inhibiting MMP activity through tissue inhibitors of metalloproteinases or synthetic MMP inhibitors can reduce BBB disruption and inflammation in traumatic brain injury models [146]. These strategies hold promise for mitigating neurovascular damage and improving outcomes in military members repeatedly exposed to blasts. Future research should focus on MMP inhibition and oxidative stress reduction as potential interventions to mitigate long-term neurological consequences in blast-exposed individuals, particularly military personnel and other at-risk populations subjected to repeated blast overpressure.

### 3.3. Blast-Induced Alterations in BBB Junctional Proteins and Vascular Endothelial Molecules

Blast exposure significantly disrupts the integrity of the BBB and the NVU, critical structures that safeguard the brain. The BBB comprises endothelial cells tightly connected by TJs, such as OCLN, CLDN-5, and ZO-1, which, along with the basement membrane, pericytes, and astrocytic endfeet, regulate the passage of substances between the bloodstream and CNS [56,83,147]. These TJs act as selective barriers, allowing essential molecules to pass while blocking harmful agents [148,149,150]. Astrocytic AQP4 channels play a complementary role by managing water balance and waste clearance through the glymphatic system [151,152]. The endothelial glycocalyx, anchored by the transmembrane proteoglycan SD-1, provides an additional protective layer, buffering against oxidative stress and controlling vascular permeability and immune cell trafficking [95,153,154]. Together, these molecular elements work synergistically to maintain BBB stability and protect the brain from neurotoxic and inflammatory processes essential for NVU health [8,155].

Our study of military breachers with career-long blast exposure revealed significant alterations in the plasma levels of key biomarkers related to BBB integrity, TJ proteins, and NVU function, including OCLN, ZO-1, AQP4, and SD-1. These findings align with the documented cumulative effects of repeated low-level blast exposure, underscoring heightened neurovascular vulnerability in this population [8,9,50,156]. Correspondingly, Agoston et al. reported that repeated low-level blast exposure in military personnel elevated serum levels of junctional proteins, such as OCLN and CLDN5, along with markers of neuronal, glial, and vascular damage that persisted up to three months post-exposure, suggesting ongoing BBB disruption with potential for long-term neurodegenerative impacts [20]. Supporting this, Ahmed et al., in a swine model, observed elevated CSF levels of OCLN and CLDN5 following blast exposure, indicative of early BBB disruption and subsequent neuroinflammatory cascades, with levels normalising within two weeks [157].

Using an animal model, Abdul-Muneer et al. demonstrated that low-level blast-induced oxidative stress causes cerebrovascular inflammation and BBB disruption, reducing CNS junctional proteins, OCLN, CLDN5, and ZO-1. This weakening of the BBB was accompanied by AQP4 activation, resulting in edema, increased permeability, and neuroinflammation [158]. Building on these findings, Heyburn and colleagues confirmed that repeated low-level blasts further compromise BBB integrity, with substantial reductions in OCLN and CLDN5, contributing to increased permeability in rats [115]. Their studies also noted regional variability in OCLN and CLDN5 expression, indicating differential susceptibility to mechanical damage across brain regions [82]. Similarly, Hue et al., in a series of in vitro and in vivo rodent studies, documented immediate BBB breakdown following blast exposure, marked by compromised ZO-1 and CLDN5, leading to increased permeability. Recovery was delayed with repeated exposures, though cumulative damage was not observed [159,160,161]. Kuriakose et al. further reported that endothelial cell degradation and TJ protein loss contribute to heightened BBB permeability post-repetitive blast exposure [71]. In thoracic blast models, Cong et al. showed that deficiency in DDAH1, a vascular repair enzyme, exacerbated oxidative stress and BBB leakage, leading to reduced levels of OCLN, CLDN5, and ZO-1, signifying greater endothelial dysfunction [142,162]. Additionally, Lucke-Wold et al. found that bryostatin-1 treatment stabilised OCLN and ZO-1 levels in the prefrontal cortex post-blast, suggesting a potential therapeutic effect on BBB integrity through protein kinase C modulation [163].

AQP4, a water channel protein primarily located in astrocytic endfeet along the BBB, is particularly vulnerable to blast-induced disruption, with significant changes in both its expression and functionality [152,164,165]. Kawoos et al. reported that blast exposure triggers an early increase in BBB permeability and elevated AQP4 expression, contributing to acute cerebral edema and neuroinflammation [54]. These findings highlight AQP4’s critical role in BBB compromise and involvement in potential repair mechanisms following blast exposure [84]. Similarly, Abutarboush et al. demonstrated that low-intensity blast exposure altered brain levels of AQP4 and glymphatic clearance pathways in rats [86]. Braun et al. determined, in both animal models and brain tissue from military veterans, that blast-induced disruption of AQP4 impairs glymphatic clearance, resulting in CNS waste accumulation and heightened neurodegenerative risk [87,166]. This mislocalisation of AQP4 aligns with broader BBB disruptions, including alterations in TJ proteins and elevated neuroinflammatory and oxidative stress responses, further compromising BBB integrity and compounding damage to the NVU [167]. Collectively, these studies highlight the dual vulnerability of cerebrovascular TJ stability and glymphatic function to repeated blast exposure, revealing pathways through which chronic BBB compromise may contribute to neurological risks in military personnel [168,169].

Elevated SD-1 levels in military breachers show its potential as a biomarker for blast-induced glycocalyx damage and dysfunction [64,92]. As an integral component of the endothelial glycocalyx, SD-1 plays a crucial role in maintaining vascular integrity and regulating BBB function, with its release into circulation marking endothelial activation or trauma-related endotheliopathy [90,170,171,172]. The elevated SD-1 detected in breachers suggests that repeated low-level blasts disrupt the glycocalyx, increasing vascular permeability and the risk of neuroinflammation, consistent with established patterns of vascular damage in blast-related neurotrauma [8,9,55]. Although direct SD-1 measurements in blast-exposed humans remain limited, supporting evidence from rodent studies by Hall et al. demonstrates that repeated low-intensity blasts compromise the glycocalyx in brain regions associated with cognitive impairment [173]. Chen et al. further showed that a single high-intensity blast induces rapid and sustained glycocalyx degradation, with SD-2 identified as a major damage marker [26]. These findings highlight syndecans and other heparan sulfate proteoglycans as valuable biomarkers and potential therapeutic targets for protecting neurovascular integrity in individuals exposed to blasts [13,47].

These reports demonstrate that repeated low-level blast exposure compromises cerebrovascular integrity by degrading critical BBB proteins, including OCLN, ZO-1, and AQP4, which are essential for maintaining TJ stability. Additionally, damage to the endothelial glycocalyx, evidenced by elevated SD-1 levels, further increases BBB permeability, amplifies neuroinflammation, and exacerbates NVU dysfunction. These findings reinforce the need for targeted interventions to mitigate cumulative neurovascular damage and reduce the long-term neurological risks associated with recurrent blast trauma.

Moving forward, it is essential to develop and implement effective countermeasures and preventive strategies for high-risk blast-exposed personnel such as military breachers. Key approaches include enhancing protective gear to mitigate blast overpressure exposure, refining operational protocols to minimise risk, and integrating real-time exposure monitoring [174,175,176,177,178]. Additionally, adopting prophylactic therapies or nutritional interventions that target oxidative stress and inflammation can help preserve BBB integrity and neurovascular function [126,179,180,181,182,183]. Early detection through biomarker surveillance, combined with timely rehabilitation and neuroprotective interventions, is crucial for limiting cumulative neurovascular injury and reducing the long-term neurological risks associated with repeated blast exposure [21,184,185].

### 3.4. Study Limitations

This study has several limitations that warrant consideration. The small sample sizes in the breacher and control groups constrain the generalizability of findings and may reduce statistical power, potentially overlooking subtle but clinically significant effects. Blast exposure history was self-reported, and direct measurements of blast overpressure were unavailable, limiting precise correlations between biomarker levels and specific exposure intensities and brain health. The cross-sectional design provides a snapshot at a single time point, underscoring the need for longitudinal studies to evaluate cumulative effects and temporal biomarker changes. Despite efforts to match participants by age, sex, and occupational stressors, unmeasured confounders—such as genetic predispositions, lifestyle factors, or other environmental exposures—may have influenced the results. The limited biomarker panel may not fully capture the intricate molecular pathways involved in neurovascular and BBB disruption; expanding the panel could provide a more comprehensive understanding of blast-related effects. Lastly, while elevated biomarker levels strongly suggest neurovascular damage and BBB compromise, this study’s design precludes causal inferences. Future longitudinal and experimental research is necessary to confirm these findings, explore mechanisms, and establish causative links between repetitive blast exposure and neurovascular injury.

## 4. Materials and Methods

All volunteers were provided with a study briefing of research activities and risks before written informed consent was obtained. The protocol was approved by the DRDC Human Research Ethics Committee (HREC: Protocol No. 2016-006) and performed in accordance with the ethical standards of the Helsinki Declaration.

### 4.1. Study Population and Experimental Design

This prospective, cross-sectional cohort study enrolled Canadian Forces School of Military Engineering (CFSME) breacher instructors and range staff (*n* = 18; hereafter, referred to as “Breachers”), each of whom was repeatedly exposed to explosive blast overpressure throughout their military careers. Unexposed sex- and age-matched Canadian Armed Forces (CAF) members served as a “Control” group (*n* = 19), who had comparable operational experience and who were routinely subject to similar physical and psychological stressors, daily work schedules, and other career demands as their breacher counterparts, other than having no history of occupational blast exposures. The direct quantification of blast overpressure was not possible in this study; however, the instructors and range staff contribute to eight to twenty breaching courses per year, with 1–2 days of breaching on the range per week, where they can be exposed to >6 blast events per day. Participants had not been exposed to blast for a minimum of 48 h and were asked to refrain from strenuous physical activity for at least 24 h prior to testing.

Data collection from each of the breachers and controls was performed during a single experimental session at the Canadian Forces Base Gagetown (CFB Gagetown, NB) training centre and Defence Research and Development Canada—Toronto Research Centre (DRDC—TRC, ON), respectively. As part of the larger multidimensional study of blast effects on military health and performance, each participant provided demographic data, underwent a comprehensive health and medical history, took occupational questionnaires including breaching and explosive experience (i.e., intensities, frequencies) and any prior concussions/brain injuries, and provided deployment-related information (e.g., type, number, and proximity of blasts), along with a self-reported symptom assessment and standardised neuropsychological tests of cognition and visuomotor coordination, auditory and vestibular function, and balance and tremors, as previously reported [17,103]. None of the participants in either group had a history of diagnosed acquired brain injury, major neurological or psychiatric disorders, learning disabilities, or current substance abuse issues.

### 4.2. Blood Sample Collection, Processing, and Storage

Peripheral venous blood was collected from participants in a fasting state by a trained technologist using standard phlebotomy techniques. Blood samples were drawn into 10 mL K2EDTA vacutainers (BD Vacutainer^®^, Franklin Lakes, NJ, USA), immediately centrifuged at 1600× *g* for 15 min at 4 °C, separated into plasma aliquots, and stored at −80 °C until analysis. All samples were processed in the same manner at the same time of day.

### 4.3. Plasma Molecular Biomarker Analyses

A panel of 10 biomarkers was selected for investigation on the basis of their potential to reflect distinct neurovascular injury mechanisms. Plasma MPO; MMP-1, -3, and -9; and MMP-2 and -10 were measured using MesoScale Discovery (MSD^®^, Gaithersburg, MD, USA) Human Ultra-Sensitive MPO single-plex (Catalog # K1534C), MMP 3-Plex (Catalog # K15034C) and MMP 2-Plex (Catalog #K15033C) MULTI-ARRAY^®^ assay kits, respectively, on a SECTOR Imager 6000 (MSD^®^). All assays were run according to manufacturer’s instructions, as previously reported [186,187].

Plasma SD-1 concentrations (ng/mL) were analysed with a commercially available quantitative immunoassay (BioVendor, LLC, Asheville, NC, USA). Absorbencies were read using an automated microplate photometer (Synergy-2 Multi-Mode Reader; BIO-TEK Instruments, Winooski, VT, USA). All test samples were analysed according to the manufacturer’s protocol, as previously reported [188,189].

Plasma concentrations of neurovascular proteins were measured using the following ELISA kits as per manufacturer’s instructions: OCLN (Novus Biologicals, Centennial, CO USA, Catalog # NBP2-80305), ZO-1 (CUSABIO, Houston, TX, USA, Catalog # CSB-E13916h), and AQP4 (CUSABIO, Houston, TX, USA, Cat # CSB-E08254h). Absorbance was read at 450 nm using Biotek Synergy-2 microplate reader (Bio Tek Instruments, Winsooski, VT, USA) [24,82].

All biomarkers were analysed neat and in duplicate, with the mean value of the duplicates used as the final value for each biomarker. Usable values were defined as those within the detection limits specified by the assay manufacturer and with a coefficient of variation (CV) <20% between duplicates. Biomarker limits of detection (LLOD) and dynamic ranges are provided on the MSD website (https://www.mesoscale.com/en). Values below the LLOD were excluded from the final analysis. To minimise variability, all samples were processed on the same day, in a random order, by a technician blinded to participant group status. The intra- and inter-assay CVs were as follows: MPO (5.5% and 5.0%), MMP-1 (6.0% and 5.4%), MMP-2 (4.5% and 5.9%), MMP-3 (6.4% and 5.2%), MMP-9 (5.3% and 4.4%), MMP-10 (5.4% and 6.9%), SD-1 (4.9% and 6.2%), OCLN (5.6% and 5.2%), and AQP4 (8% and 10%).

### 4.4. Statistical Analysis

For all data collected, an outlier analysis and normality and variance assumptions checks were performed. Continuous variables are expressed as mean ± standard deviation (±SD) if normally distributed or median and interquartile range (IQR) if non-normally distributed. Categorical variables are shown as frequencies (%). Independent-sample *t*-tests for normally distributed variables and non-parametric Mann–Whitney *U*-tests for non-normally distributed quantitative variables were used to evaluate differences between two groups. Individual biomarker values were deemed valid for statistical quantitation if replicate values for each biomarker displayed a coefficient of variation (CV) below 20% and fell within the manufacturer’s recommended limits of detection. Statistical significance was assessed at a false discovery rate (FDR) corrected *p* < 0.05 (two-tailed). Analyses were performed using Prism 10 (GraphPad Software, LLC, version 10.2.3, San Diego, CA, USA).

## 5. Conclusions

Our study provides direct evidence that military personnel with repeated low-level blast exposure, such as breachers, have elevated plasma levels of critical biomarkers associated with neurovascular and BBB dysfunction, including MPO, MMP-3, MMP-9, MMP-10, OCLN, and SD-1. These results underscore the cumulative impact of blast exposure on neurovascular integrity via oxidative stress, inflammation, and extracellular matrix degradation, which collectively allows neurotoxic substances to enter the brain parenchyma. In line with findings from animal and other human studies, our data confirm that heightened MPO and MMP activity disrupt the BBB and amplify neuroinflammation, leading to immediate and long-term neurological consequences. This study highlights the significant neurovascular risks posed by occupational blast exposure and points to the potential for targeted therapies to mitigate oxidative and inflammatory damage. These findings pave the way for enhanced protective measures and intervention strategies to safeguard brain health in military personnel exposed to repeated low-level blasts.

## Figures and Tables

**Table 1:** Demographics and service history.

Variables	Breachers (*n* = 18)	Controls (*n* = 19)	Mean Difference (95% CI)	Cohen’s D	*p* *
Age (yrs)	33 (27–38)	32 (27.5–35.5)	0.8 (−4.4–5.7)	0.16	0.742
Sex (n, % male)	17 (89.5)	17 (89.5)	0 (−21.1–21.1)	−0.02	0.790
Military service (yrs)	11.3 (9–14.5)	5 (1.5–10.2)	6.4 (3.2–10.3)	1.6	**<0.001**
Exposure to explosives (yrs)	10 (7.5–12)	0 (0–0)	10.4 (8–13.1)	3.5	**<0.001**
Breaching (yrs)	7 (4.5–10)	0 (0–0)	7.1 (5.2–9.3)	3.5	**<0.001**
Combat deployment	11 (64.7)	0 (0)	68.2 (47.4–89.5)	2.9	**<0.001**
**Status**					
Regular Force	9 (47.4)	8 (42.1)	5.8 (−21.1–31.6)	0.16	0.546
Reservist	10 (52.6)	11 (57.9)	5.8 (−21.1–31.6)	0.16	0.546
**Rank**					
Junior NCM	5 (26.3)	13 (68.4)	−42.4 (−68.4–−10.5)	−1.3	**0.004**
Senior NCM	12 (63.2)	0 (0)	63 (42–84.5)	2.6	**<0.001**
Junior Officer	2 (10.5)	6 (31.6)	−20.6 (−47.4–10.5)	−0.76	0.098

Notes. Continuous/integer data presented as median and interquartile range—med (IQR); categorical data presented as the frequency and percent = *n* (%). NCM = Non-commissioned member. * Significance determined by independent two-sample *t*-test or chi-squared (Χ^2^) test for breachers vs. controls, with a false discovery rate correction set at *p* = 0.05 (**bold *p*-values**). For categorical variables, the mean differences were assessed based on the percentage of individuals classified within each outcome category.

**Table 2:** History of prior head injury.

Variables	Breachers (*n* = 18)	Controls (*n* = 19)	Mean Difference (95% CI)	Cohen’s D	p *
Concussion	8 (44.4)	5 (26.3)	21 (−5.3–47.4)	0.64	0.088
Physical impact to head	9 (47.4)	11 (57.9)	−10.4 (−36.8–15.8)	−0.30	0.402
MVA	14 (73.7)	9 (47.4)	2.6 (−5.3–52.6)	0.78	0.066
Fallen as child	8 (42.1)	6 (31.6)	10.4 (−10.5–31.6)	0.31	0.206
Physical fight	13 (68.4)	15 (78.9)	−10.6 (−31.6–15.8)	0.35	0.258
Blast exposure	19 (100)	2 (10.5)	89.2 (73.7–100)	6.3	**<0.001**

Notes. MVA = motor vehicle accident. Data presented as frequency and (%). * Significance determined using independent two-sample chi-squared (Χ^2^) test for breachers vs. controls, with a false discovery rate correction set at *p* = 0.05 (**bold *p*-values**), derived from bootstrapped mean difference testing evaluated on percent of individuals categorised to each outcome.

**Table 3:** Neurocognitive and neuropsychological measures.

Variables	Breacher (*n* = 18)	Controls (*n* = 19)	Bootstrap	p *
**Neuropsychological Measures**				
**RAND SF-36**				
General health	75 (67.5–80)	80 (65–92.5)	0.7	0.478
Physical functioning	95 (92.5–100)	100 (95–100)	1.6	0.120
Emotional well-being	80 (66–88)	84 (68–88)	0.4	0.676
Social functioning	100 (87.5–100)	100 (7–100)	0.3	0.786
Pain	90 (80–90)	90 (85–100)	1.5	0.126
Energy	50 (37.5–67.5)	65 (60–80)	2.2	**0.022**
**Rivermead**				
RPQ3	2 (1–5.5)	0 (0–2)	3.8	**<0.001**
RPQ13	7.0 (1.5–15.5)	0 (0–2.5)	4.0	**<0.001**
Somatic	0.6 (0.1–1.1)	0 (0–0.1)	3.6	**0.004**
Cognitive	0 (0–1.3)	0 (0–0.1)	2.9	**0.004**
Emotional	0 (0–0.9)	0 (0–0.1)	3.4	**<0.001**
**Neurocognitive Measures**				
Four-choice RT task (ms)	451 (406–519.5)	450 (425.5–517)	0.7	0.538
**n-back (d′)**				
1-back	4.7 (3.8–4.7)	4 (3.4–4.7)	1.2	0.218
2-back	2.5 (2.1–3.1)	2.8 (2.2–3.5)	0.7	0.490
3-back	1.2 (0.9–1.6)	1.3 (1.2–1.7)	1.1	0.254
dMTS (% correct)	68 (56–78)	72 (66–82)	1.6	0.094
Stroop (ms)	48 (16.2–80.2)	49 (38–62.5)	0.1	0.918

Notes: Interval and continuous data presented as median and interquartile range (IQR); categorical data presented as frequency and percent—*n* (%). RT, reaction time; ms, milliseconds; MTS, delayed matching-to-sample task. For n-back and dMTS numbers indicate accuracy (%); Stroop numbers indicate reaction time (s). Significance corrected at a false discovery rate *p = 0.05 (**bold *p*-values**), derived from bootstrapped mean difference testing.

## Data Availability

The datasets supporting the findings of this article are included within the article itself. However, due to the sensitive nature of the data, which could potentially compromise the privacy of military research participants, the full datasets are not publicly available. Requests for access to additional datasets can be directed to the corresponding author.
